# Supplementary material for: GLP-1 receptor agonists’ impact on cardio-renal outcomes and mortality in T2D with acute kidney disease
Source: Nat Commun. 2024 Jul 13;15:5912. doi: 10.1038/s41467-024-50199-y (PMC11246471; doi:10.1038/s41467-024-50199-y)
Supplement: Supplementary file 1 — Supplementary Information [file 41467_2024_50199_MOESM1_ESM.pdf]

Supplementary Information to

GLP-1 Receptor Agonists' Impact on Cardio-Renal Outcomes and Mortality in T2D with Acute Kidney Disease

(A) Supplementary material and methods:

Introduction to the TriNetX database

External validation

Sensitivity analyses, specificity, positive outcome controls, and negative outcome controls

Methodological divergence and outcome analysis between the current study and our previous SGLT-2 Inhibitors study using the same TriNetX database

(B) Outcome definition

(C) Supplementary table

Suppl. Table 1. Presumptive causes of AKI

Suppl. Table 2. Renal function and electrolytes post withdrawal of dialysis

Suppl. Table 3. Incidence rate ratios and E-values of outcomes of interest among the GLP-1 RAs users compared to the control group after propensity score matching

Suppl. Table 4. Risk of mortality in type 2 diabetes patients with AKD: comparison between GLP-1 RAs users and non-users after propensity score matching

Suppl. Table 5. Risk of MACE in type 2 diabetes patients with AKD: comparison between GLP-1 RAs users and non-users after propensity score matching

Suppl. Table 6. Risk of MAKE in type 2 diabetes patients with AKD: comparison between GLP-1 RAs users and non-users after propensity score matching

Suppl. Table 7. Sensitivity analysis for all-cause mortality, MACE, and MAKE between GLP-1 RAs users and non-users

Suppl. Table 8. Sensitivity analysis for incidence of outcomes of interest among GLP-1 RAs users compared to non-users after propensity score matching in patients with eGFR ≥ 30 ml/min/1.73m^2 treated with Exenatide

Suppl. Table 9. Sensitivity analysis for incidence of outcomes of interest among GLP-1 RAs users compared to non-users after propensity score matching in patients with eGFR < 30 ml/min/1.73m^2 treated with Exenatide

Suppl. Table 10. Sensitivity analysis for all-cause mortality, MACE, and MAKE between GLP-1 RAs users and other second-line antihyperglycemic treatments users (Sulfonylureas, dipeptidyl peptidase-4 inhibitor or Pioglitazone) user in a new-user design

(D) Supplementary figure

Suppl. Figure 1. Comparative HbA1C mean levels at baseline, D0-30, and D60-90 for GLP-1 RAs users and the control group

Suppl. Figure 2. Comparative eGFR mean levels at baseline, D0-30, and D60-90 for GLP-1 RAs users and the control group

Suppl. Figure 3. Comparative HbA1C mean levels at baseline, D0-30, and D60-90 for GLP-1 RAs users and other second-line antihyperglycemic treatments users

Suppl. Figure 4. Sensitivity, specificity, positive outcome controls, and negative outcome controls

Suppl. Figure 5. Graphic abstract

Suppl. Figure 6. External validation by CGRD database. The Kaplan-Meier curves presented the long-term outcomes of interest, including (A) MACE (B) MAKE

(E) Supplementary reference

(G) STROBE statement checklist

(A) Supplementary material and methods:

Introduction to the TriNetX database

1. TRINETX FEDERATED DATA NETWORK

TriNetX was initially developed with the aim of making collaborative industry–academia clinical trial research more efficient. It enables researchers to use real-world data to design trials that have the potential to meet their accrual requirements, and to identify performance sites that should be invited to open a trial. Beginning in 2015, TriNetX contacted health care organizations (HCOs) that had established i2b2 research repositories to join the network as data providers. Over the years, the data harmonization processes within TriNetX have evolved and improved, removing the requirement for data sources to have an i2b2 repository. TriNetX functions on a hub-and-spoke model, wherein Pharma and CRO sponsors pay a subscription fee to query for aggregate counts from the HCOs in the network, which are populated with deidentified patient data. This business model has proven successful, with 14 leading Pharma and CRO sponsors subscribed, and 79 HCO data providers in the network.[1]

2. NETWORK INFRASTRUCTURE

TriNetX is a multitenant software-as-a-service platform that utilizes Amazon Web Services (AWS) for its architecture, as depicted in Figure 1. Health Care Organizations (HCO) data accessible through the TriNetX network is stored on the appliance that is located at each HCO data center. During the onboarding process, the data is loaded onto the appliance with an extract-transform-load process that leverages the existing capabilities and scripting of the TriNetX agent. In addition to i2b2, TriNetX supports the loading of data from other source systems with a combination of its product and service capabilities [1].

3. SECURITY

TriNetX is deployed on a secure Health Insurance Portability and Accountability Act-compliant (HIPAA) virtual private cloud hosted by AWS. This cloud meets Federal Risk and Authorization Management Program (FedRAMP), NIST 800-53, and other industry-standard security certifications. Access to TriNetX is secured with Transport Layer Security (TLS) and a 2048-bit security certificate. Services hosted behind AWS's Elastic Load Balancer are configured using the AWS Elastic Load Balancer Security Policy 2015-05. (https://aws.amazon.com/security/)

The TriNetX appliance is highly secure and locked down with no extraneous processes running. All communication is initiated outbound, and penetration and vulnerability tests are regularly conducted against the hosted application environment. Expert attestation of the appliance's security is available to TriNetX members, with full documentation available.[1] TriNetX is the global federated health

research network providing access to electronic medical records (diagnoses, procedures, medications, laboratory values, genomic information) across large healthcare organizations (HCOs). This report was run on the set of HCOs grouped into a network called Research. This network included 76 HCO(s).

4. CLINICAL DATA, CONTROLLED TERMINOLOGIES, AND SEMANTIC MAPPING

TriNetX is an innovative healthcare network that provides demographic, diagnosis, procedure, medication, and laboratory data for research purposes. In addition, TriNetX has recently expanded their offering to include tumor registry and molecular genomic data. Furthermore, they plan to add vital signs and other observation data relevant to oncology and pulmonology in the near future. This will provide researchers with a comprehensive and real-world dataset of clinical data to facilitate evidence-based research. The data ingested by the TriNetX appliance varies in origin depending on the healthcare organization (HCO). Some HCOs extract data directly from their electronic health records (EHRs), while others have data warehouses with varying common data models, [2] such as i2b2 and observational health data sciences and informatics. A typical commercial EHR uses a myriad of proprietary code system standards or terminology standards that may vary by country, such as the United States' Clinical Modification version of the International Classification of Diseases (ICD), Tenth Revision. In the United States, procedures are coded using ICD-10-PCS and Current Procedural Terminology (CPT), yet there is no accepted standard for procedures in other countries. Many EHRs incorporate proprietary drug data, including First DataBank, Wolters Kluwer’s Medi-Span, and Cerner’s Multum, each of which has a different identifier for the same drug. Medications may also be coded to national drug codes or to anatomic therapeutic chemical codes, used in many European countries, or local codes. Additionally, laboratory information systems at HCOs and commercial laboratories rarely use standard codes, such as Logical Observation Identifiers Names and Codes (LOINCs), for test results.

5. DATA QUALITY

Data quality is a major challenge when it comes to the proper use of research data, and can potentially compromise the validity of research results.[3] Although the adoption of Electronic Health Records (EHRs) has grown exponentially due to federal incentives and meaningful use requirements, the quality of the data within them, and therefore the data used for research, is still improving. As EHRs are primarily designed for billing and patient care functions, the data may not be of the highest quality for research purposes.16 To address this issue, a comprehensive data-quality framework and approach are needed.[4] Research on data quality is limited and generally focuses on assessing the quality of data in a single system or institution, [5]as well as determining if the data is good enough for its primary purpose, which is providing clinical care to patients. TriNetX has developed a comprehensive methodology to assess the quality of the data it uses. This methodology, which consists of four Cs—cleanliness, consistency, correctness, and completeness—takes into account the

data extracted from the source systems, which is then transformed, cleaned-up, deduplicated, de-identified, optionally obfuscated, and semantically mapped.

6. ANALYSIS SPECIFICATIONS

The Compare Outcomes Analytic offers four different types of analyses: Measure of Association, Survival, Number of Instances, and Lab Result Distribution. The first three analyses have the option to "exclude patients with outcomes prior to the window." This option is useful when analyzing outcomes that are chronic diseases, as patients who have already developed the outcome are not at risk of developing it during the time window. When the "exclude patients with outcomes prior to the time window" option is unchecked, all patients in the cohort will be included in the analysis, regardless of whether they had the outcome prior to the time window. However, if this option is checked, patients will be excluded if their medical record indicates that they had the outcome before the start of the time window. This exclusion will apply to all patients who had the outcome prior to the index event, and any patients who develop the outcome between the index event and the start of the time window will also be excluded if the time window starts some days after the index event.

7. MEASURE OF ASSOCIATION ANALYSIS

The Measure of Association Analysis assesses the fraction of patients with a specified outcome. The output summary includes the number of patients in each cohort meeting the query criteria, the number of patients with the outcome in each cohort, and the risk of the outcome in each cohort. We made adjustments for various factors including demographics (age, sex, race), health conditions (underlying chronic kidney disease (CKD), hyperuricemia, congestive heart failure, ischemic heart diseases, cerebrovascular diseases, overweight, chronic obstructive pulmonary disease, musculoskeletal disease, malignancy), medications (Metformin, Sulfonylureas, Acarbose, Insulin, Aspirin, Clopidogrel, Atrovastatin, Allopurinol, Febuxostat, alpha-blocker, beta-blocker, calcium channel blocker), and clinical metrics (body mass index, leukocyte count, platelet count, estimated glomerular rate, proteinuria level, total cholesterol, glycohemoglobin, aspartate transaminase, B-type natriuretic peptide). Furthermore, the output includes the Risk Difference [the difference in the risks in cohorts of type 2 diabetes with acute kidney disese (AKD) who received and not received glucagon-like peptide-1 receptor agonists (GLP-1 RAs)], Risk Ratio (the ratio of the risks in GLP-1 RAs users cohort and non-user cohort), and Odds Ratio (the ratio of the odds in GLP-1 RAs users cohort and non-user cohort).

8. SURVIVAL ANALYSIS

The Kaplan-Meier Analysis estimates the probability of the outcome at a respective time interval (in this analysis, daily time intervals are used). To account for patients who exited the cohort during the analysis period, censoring is applied, whereby these patients are removed from the analysis after the last fact in their record. The output summary includes the number of patients in

each cohort (meeting the query criteria), the number of patients with the outcome in the time window, median survival (the number of days when survival drops below 50%; the "-" indicates survival does not drop below 50% during the time window), and survival probability at the end of the window (the % survival at the end of the window). Furthermore, the Log-Rank Test, Hazard Ratio, and test for Proportionality are employed.

9. NUMBER OF INSTANCES ANALYSIS

The Number of Instances Analysis is a method that calculates the frequency of an outcome within a given time window. This analysis includes two settings: patients with zero instances and the definition of an instance. When selecting to exclude patients with zero instances, these patients are not included in the calculations for mean number of instances, standard deviation, or median. The histogram that displays the distribution of patients by number of instances will not include a bar for zero. Alternatively, by selecting to include patients with zero instances, the mean, standard deviation, and median for the number of instances will reflect the entire patient population, including those with zero instances. The histogram will include a bar for zero instances. The definition of an instance impacts how the counts are analyzed. When selecting Date, each calendar date on which any of the terms selected in the outcome are recorded will represent one instance. For instance, if the outcome is "Med A or Med B," and a patient has "Med A" on January 3, both medications on January 4, and "Med B" on January 6, then that patient is considered to have three instances, representing January 3, January 4, and January 6. It is important to note that if an outcome occurs across multiple dates, only the start date is tracked for the purpose of counting instances. For instance, a patient who begins a hospital stay on January 1, ends on January 3, begins another stay on January 10, and ends on January 15, is considered to have two instances of the outcome. Selecting Visit as an instance will count any visit that includes the outcome as one instance, regardless of how many times it occurred. For example, a patient administered an analgesic on each of the three days of an inpatient stay following some index event. If analgesic is an outcome, these three administrations will represent only one instance because they are associated with the same visit. The output summary includes the count of patients in the cohort, the count of patients in the cohort that had the outcome in the time window, the mean, standard deviation, and median of the counts, and the median (1+ instances) when patients with zero instances are included in the analysis. Additionally, T-Test statistics testing for the difference between the cohorts are included.

10. LABORATORY RESULTS ANALYSIS

In the analysis, only lab results that are relevant to the outcomes are included. Furthermore, only the most recent lab values within the time window are taken into account. For numeric lab results, the outcome summary contains the number of patients in the cohort who meet the query criteria, the number of patients with the outcome within the time window, the mean, and the standard deviation of the lab values in the cohort. Additionally, T-Test statistics are provided to evaluate the difference

between the cohorts. For non-numeric lab results, the counts of Negatives, Positives, and Unknowns are reported, and these percentages of the total counts are represented in the form of a bar chart.

11. LIMITATION

The TriNetX platform operates on individual-level data. However, as researchers, we do not have direct access to this individual data. Instead, the platform provides us with aggregated counts and statistical summaries of de-identified data, ensuring the privacy and confidentiality of the individuals represented in the data. The TriNetX platform pools data from various participating institutions, and while it processes individual-level data, it only allows researchers like us to interact with and analyze the aggregated and summarized data. This ensures compliance with both the Health Insurance Portability and Accountability Act and the General Data Protection Regulation.

External validation

We utilized data from the Chang Gung Research Database (CGRD), which is the most extensive collection of electronic medical records (EMR) across multiple institutions in Taiwan[6]. This database provides researchers with convenient access to standardized patient-level data, enabling efficient utilization for a range of studies related to GLP-1 RAs. Investigations assessing the beneficial effects and potential adverse side effects of GLP-1 RAs have relied on this resource[7-11]. The CGRD is a comprehensive collection of daily medical records gathered prospectively from seven branches of Chang Gung Memorial Hospital in Taiwan since January 2001. This database encompasses a significant volume of medical information, with an average of 500,000 emergency department visits, 8,500,000 outpatient visits, and over 280,000 admissions to 10,070 beds annually[6]. The CGRD includes detailed personal information about patients, such as gender, body weight, height, lifestyle, and birth date. It also contains laboratory findings, pathology reports, imaging exam results, and comprehensive information about every emergency, inpatient, and outpatient visit. The database uses International Classification of Diseases, 9th and 10th revision, Clinical Modification (ICD-9-CM and ICD-10-CM) codes for classifying underlying diseases, reasons for admission, and details of emergency and outpatient visits. To prioritize patient privacy, the chart number of each patient was encrypted and exclusively utilized for data linkage between different databases within the CGRD[12]. Our study received approval from the Institutional Review Board of the Chang Gung Medical Foundation (IRB No.: 201702274B0), ensuring adherence to ethical guidelines and patient confidentiality. The CGRD's extensive collection of medical information made it an optimal resource for conducting retrospective clinical studies, greatly facilitating our research efforts.

Sensitivity, specificity, positive outcome controls, and negative outcome controls

To ensure the reliability of our findings, several sensitivity analyses were carried out. Firstly, we investigated the relationship between variables across different enrollment periods to account for possible changes in antihyperglycemic prescribing preferences over time. Additionally, we excluded patients with short follow-up durations and those who experienced mortality at different times during the follow-up period. Our study utilized global healthcare data from TriNetX, spanning from September 2002 to December 2022. Notably, considering that GLP-1RAs were not introduced until April 2005, we conducted a sensitivity analysis by adjusting the start point of our dataset to 2006[13]. Furthermore, with the FDA's approval of the GLP-1 RAs (Liraglutide) for weight loss in September 2014, we conducted a distinct sensitivity analysis to address potential selection bias for patients in the non-GLP-1 RAs group before 2015[14]. Moreover, we incorporated subjects who experienced events within 90 days post-discharge during the AKD phases in another sensitivity analysis. Importantly, sensitivity analyses were also performed for patients with severe renal insufficiency (eGFR < 30 ml/min/1.73m^2) prescribed exendin-based GLP-1RAs, to scrutinize the safety and outcomes of these medications in a high-risk population, following current clinical recommendations[15,16]. Secondly, diverse covariates were included in the Cox regression models to further validate the robustness of our results within each cohort. Thirdly, we utilized a new-user design focusing on those newly initiating GLP-1 RAs and juxtaposed this with individuals newly starting other second-line antihyperglycemic treatments, namely Sulfonylureas, dipeptidyl peptidase-4 inhibitors, or Pioglitazone. Furthermore, specificity analyses were performed to examine the beneficial effects of GLP-1 RAs on different composite adverse outcomes, including 3-point MAKEs (redialysis, dialysis dependence, or eGFR < 15 ml/min/1.73 m2), mortality with myocardial infarction, and mortality with stroke.

To evaluate the effectiveness of our approach in reproducing known associations, we tested nausea as positive outcome control[17,18]. Additionally, we explored the correlation between GLP-1 RAs treatment and seven unrelated events (conjunctivitis, melanoma, fracture, traffic accidents, osteosarcoma, lupus, and Crohn's disease) as specified negative outcome controls. No prior evidence suggested a causal relationship between GLP-1 RAs and the specified negative outcome controls. Sensitivity, specificity analysis, positive outcome controls and negative outcome controls were performed using R software (version 3.2.2, Free Software Foundation, Inc, Boston, MA), SAS software (version 9.2, SAS Inc., Cary, NC), and Stata/MP software (version 16, StataCorp, College Station, TX) s part of our rigorous analytical approach.

Methodological divergence and outcome analysis between the current study and our previous sodium-glucose cotransport protein 2 (SGLT-2) inhibitors study using the same TriNetX database

Our earlier investigation into the effects of SGLT-2 inhibitors, utilizing the TriNetX database, demonstrated the value of this resource for studying novel anti-hyperglycemic agents[19]. In the current study, we have introduced key methodological differences, particularly concerning the definitions and analyses of secondary outcomes. Notably, we have replaced cardiogenic shock with cardiogenic arrest in the criteria for major adverse cardiac events (MACE) and added “eGFR < 15 ml/min/1.73m^2”to our criteria for major adverse kidney events (MAKE) to better reflect outcomes from GLP-1 RAs research[20-22]. These changes aim to enhance the specificity and clinical applicability of our findings. The decision to implement a four-point MAKE criterion, especially the inclusion of “eGFR < 15 ml/min/1.73m^2”, has led to the exclusion of patients who would have been included under the three-point MAKE definition used in our previous study. This was a strategic move to hone in on the impacts of acute kidney injury (AKI), distinct from CKD progression, thereby ensuring a clearer analysis of GLP-1 RA's effects post-AKI.

The notable decrease in mortality risk seen in the GLP-1 RAs group, when compared to the SGLT-2 inhibitors group, is likely due to variations in baseline comorbidities and demographics. The GLP-1 RAs cohort was younger and had a lower incidence of CKD, heart failure, and ischemic heart disease, which may account for the observed increase in survival. The exclusion of patients with advanced CKD from the current study aimed to reduce confounding and allowed for a more homogeneous population, thereby highlighting the potential acute benefits of GLP-1 RAs treatment.

In conclusion, the observed difference in mortality reduction between the GLP-1 RA group in the current study and the SGLT-2 inhibitors group from our earlier investigation can be attributed not only to differences in cohort size but also to methodological and demographic variations. These findings add to the evidence of the extensive benefits of GLP-1 RAs and underscore the importance of continued research to clarify these drugs' role in the clinical management of diabetes and its related complications.

(B) <u>Outcome definition</u>

**Mortality**		
**Outcome definition**		
Demographics	Deceased	Deceased
Diagnosis	UMLS:ICD10CM:R99	Ill-defined and unknown cause of mortality
Diagnosis	UMLS:ICD10CM:R99-R99	Ill-defined and unknown cause of mortality (R99)
Diagnosis	UMLS:ICD10CM:R69	Illness, unspecified

**MACE**		
**Outcome definition**		
Diagnosis	UMLS:ICD10CM:I63	Cerebral infarction
Diagnosis	UMLS:ICD10CM:I61	Nontraumatic intracerebral hemorrhage
Demographics	Deceased	Deceased
Diagnosis	UMLS:ICD10CM:R99	Ill-defined and unknown cause of mortality (R99)
Diagnosis	UMLS:ICD10CM:R69	Illness, unspecified
Diagnosis	UMLS:ICD10CM:I46	Cardiac arrest
Diagnosis	UMLS:ICD10CM:I21	Acute myocardial infarction

**MAKE**		
**Outcome definition**		
Demographics	Deceased	Deceased
Diagnosis	UMLS:ICD10CM:R99	Ill-defined and unknown cause of mortality
Diagnosis	UMLS:ICD10CM:R99-R99	Ill-defined and unknown cause of mortality (R99)
Diagnosis	UMLS:ICD10CM:R69	Illness, unspecified
Procedure	UMLS:CPT:1012740	Dialysis Services and Procedures
Procedure	UMLS:CPT:90945	Dialysis procedure other than hemodialysis (eg, peritoneal dialysis, hemofiltration, or other continuous renal replacement therapies), with single evaluation by a physician or other qualified health care professional
Diagnosis	UMLS:ICD10CM:N18.6	End stage renal disease

Diagnosis	UMLS:ICD10CM:Z99.2	Dependence on renal dialysis
Laboratory	TNX:8001	Glomerular filtration rate/1.73m^2 predicted [Volume Rate/Area] in Serum, Plasma or Blood by Creatinine-based formula (MDRD) (between 5.00 and 15.00 mL/min/1.73m^2 (most recent occurrence)

**Accidental poisoning**		
**Outcome definition**		
Diagnosis	UMLS:ICD10CM:T39	Poisoning by, adverse effect of and underdosing of nonopioid analgesics, antipyretics and antirheumatics
Diagnosis	UMLS:ICD10CM:T42	Poisoning by, adverse effect of and underdosing of antiepileptic, sedative- hypnotic and antiparkinsonism drugs
Diagnosis	UMLS:ICD10CM:T38	Poisoning by, adverse effect of and underdosing of hormones and their synthetic substitutes and antagonists, not elsewhere classified
Diagnosis	UMLS:ICD10CM:T40	Poisoning by, adverse effect of and underdosing of narcotics and psychodysleptics [hallucinogens]
Diagnosis	UMLS:ICD10CM:T51	Toxic effect of alcohol
Diagnosis	UMLS:ICD10CM:T59	Toxic effect of other gases, fumes and vapors
Diagnosis	UMLS:ICD10CM:T56	Toxic effect of metals
Diagnosis	UMLS:ICD10CM:T57	Toxic effect of other inorganic substances
Diagnosis	UMLS:ICD10CM:T54	Toxic effect of corrosive substances

**Atopic dermatitis**		
**Outcome definition**		
Diagnosis	UMLS:ICD10CM:L20	Atopic dermatitis
Diagnosis	UMLS:ICD10CM:L20-L30	Dermatitis and eczema

**Conjunctivitis**		
**Outcome definition**		
Diagnosis	UMLS:ICD10CM:H16	Keratitis

Melanoma in situ		
**Outcome definition**		
Diagnosis	UMLS:ICD10CM:D03	Melanoma in situ

(C) Supplementary table

Suppl. Table 1. Presumptive causes of AKI

	**All patients (n=165860)**	**GLP-1 RAs group (n=7511)**	**Control group (n=158349)**	**Std diff**
Cardiogenic shock	10921 (6.6%)	627 (8.3%)	10294 (6.5%)	0.03
Cardiorenal syndrome	56674 (34.2%)	2885 (38.4%)	53789 (34.0%)	0.02
Sepsis without septic shock	91555 (55.2%)	4842 (64.5%)	86713 (54.8%)	< 0.01
Septic shock	28288 (17.1%)	1533 (20.4%)	26755 (16.9%)	0.03
Hypovolemic shock	4433 (2.7%)	127 (1.7%)	4306 (2.7%)	0.06
Obstructive uropathy	12971 (7.8%)	745 (9.9%)	12226 (7.7%)	< 0.01
Drug-related AKI or Contrast nephropathy	11991 (7.2%)	697 (9.3%)	11294 (7.1%)	0.06
Others*	9303 (5.6%)	561 (7.5%)	8742 (5.5%)	0.03

Abbreviation: AKI, acute kidney injury; GLP-1; glucagon-like peptide; Std diff, Standardized difference

*Others: hypertension crisis, postpartum AKI, etc

Suppl. Table 2. Renal function and electrolytes post withdrawal of dialysis

	GLP-1 RAs group (n=7511)	Control group (n=158349)	Std diff
eGFR, mL/min/1.73m2	70.5 ± 32.7	69.4 ± 36.1	0.05
Sodium, mEq/L	138.0 ± 3.3	138.0 ± 3.6	0.01
Potassium, mEq/L	4.1 ± 0.5	4.1 ± 0.5	0.08

Abbreviation: eGFR, estimated glomerular filtration rate; GLP-1; glucagon-like peptide; Std diff, Standardized difference

Suppl. Table 3. Incidence rate ratios and E-values of outcomes of interest among the GLP-1 RAs users compared to the control group after propensity score matching

Outcome	Total	Event	Mean follow up years	Incidence rate /1000 PY	Incidence rate ratio (95% CI)	E-value (lower limit of CI)
**Mortality**	14984	1476	4.3	22.91	0.50 (0.45-0.56)	2.90 (3.32)
GLP-1RAs group	7492	508	4.4	15.41		
Control group	7492	968	4.2	30.76		
**MACE**	10485	1759	4.0	41.94	0.77 (0.70-0.85)	1.54 (1.92)
GLP-1RAs group	5251	777	4.0	36.99		
Control group	5234	982	3.9	48.11		
**MAKE**	12991	1735	4.2	31.80	0.66 (0.60-0.73)	2.09 (2.39)
GLP-1RAs group	6513	701	4.2	25.63		
Control group	6478	1034	4.1	38.93		

Abbreviations: CI, confidence interval; GLP-1 RAs; glucagon-like peptide-1 receptor agonists; MACE, major adverse cardiac events; MAKE, major adverse kidney events; PY, person-years

Suppl. Table 4. Risk of mortality in type 2 diabetes patients with AKD: **comparison between GLP-1 RAs users and non-users after propensity score matching.** The results presented in the table below are from an analysis of a cohort of patients after propensity score matching. Statistical analysis was performed using the two-sided log-rank test and risk analysis, with no adjustments for multiple comparisons. The table provides detailed statistical results, including test statistics (e.g., z, χ^2), confidence intervals, effect sizes (hazard ratios, risk differences, risk ratios, odds ratios), degrees of freedom, exact P values and 95% confidence intervals.

Mortality

Risk analysis

Cohort		Patients in cohort	Patients with outcome	Risk
1	(new)DM+AKDwithGLP-1	7,492	508	0.068
2	(new)DM+AKDwithoutGLP-1	7,492	968	0.129

		95% CI	z	p
**Risk Difference**	-0.061	(-0.071, -0.052)	-12.611	0.000
**Risk Ratio**	0.525	(0.474, 0.581)	N/A	N/A
**Odds Ratio**	0.490	(0.438, 0.549)	N/A	N/A

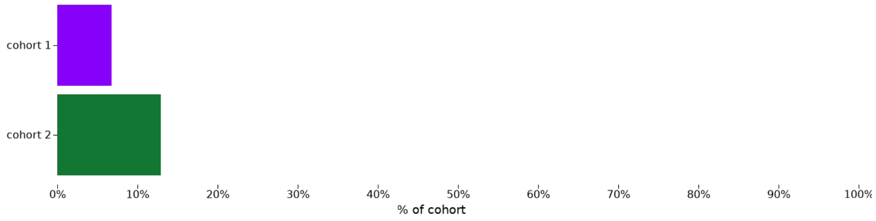

Kaplan - Meier survival analysis

Cohort	Patients in cohort	Patients with outcome	Median survival	Survival probability at end of time window

			(days)	
1 (new)DM+AKDwithGLP-1	7,492	508	--	86.54%
2 (new)DM+AKDwithoutGLP-1	7,492	968	--	78.40%

	χ^2	df	p
**Log-Rank Test**	107.959	1	0.000

	Hazard Ratio	95% CI	χ^2	df	p
**Hazard Ratio and Proportionality**	0.570	(0.512, 0.635)	2.696	1	0.101

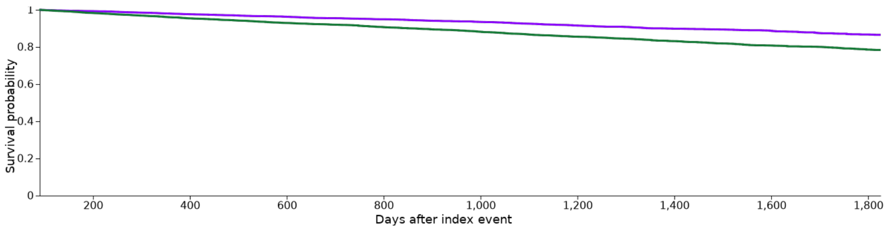

**Number of instances**					
Cohort	Patients in cohort	Patients with outcome	Mean	Standard Deviation	Median
1 (new)DM+AKDwithGLP-1	7,492	508	1.220	0.611	1
2 (new)DM+AKDwithoutGLP-1	7,492	968	1.237	0.558	1

	t	df	p
**Test Statistics**	-0.510	1474	0.610

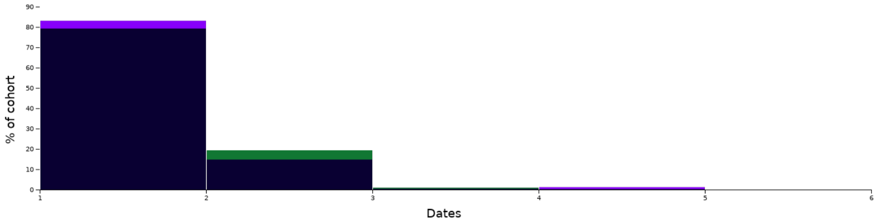

3 data points for Cohort 1 and 3 data points for Cohort 2 were omitted for display purposes.

Suppl. Table 5. Risk of MACE in type 2 diabetes patients with AKD: comparison between GLP-1 RAs users and non-users after propensity score matching. The results presented in the table below are from an analysis of a cohort of patients after propensity score matching. The baseline cohort included patients with a history of MACE, who were excluded from the analysis. Statistical analysis was performed using the two-sided log-rank test and risk analysis, with no adjustments for multiple comparisons. The table provides detailed statistical results, including test statistics (e.g., z, χ^2), confidence intervals, effect sizes (hazard ratios, risk differences, risk ratios, odds ratios), degrees of freedom, exact P values and 95% confidence intervals.

MACE

Risk analysis excluding patients with outcome prior to the time window

Cohort		Patients in cohort	Patients with outcome	Risk
1	(new)DM+AKDwithGLP-1	5,251	777	0.148
2	(new)DM+AKDwithoutGLP-1	5,234	982	0.188

		95% CI	z	p
**Risk Difference**	-0.040	(-0.054, -0.025)	-5.432	0.000
**Risk Ratio**	0.789	(0.724, 0.859)	N/A	N/A
**Odds Ratio**	0.752	(0.678, 0.834)	N/A	N/A

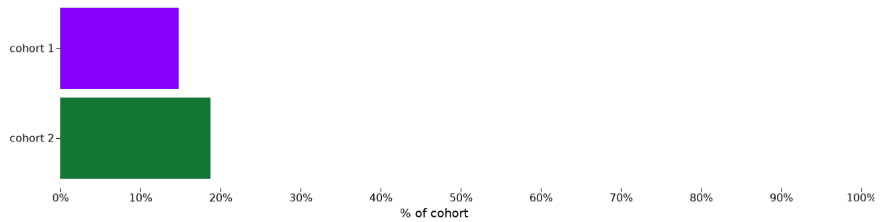

2,241 patients in Cohort 1 and 2,258 patients in Cohort 2 were excluded from results because they had the outcome prior to the time window.

Kaplan - Meier survival analysis excluding patients with outcome prior to the time window

Cohort		Patients in cohort	Patients with outcome	Median survival (days)	Survival probability at end of time window
1	(new)DM+AKDwithGLP-1	5,251	777	--	71.96%
2	(new)DM+AKDwithoutGLP-1	5,234	982	--	69.92%

	χ^2	df	p
**Log-Rank Test**	7.439	1	0.006

	Hazard Ratio	95% CI	χ^2	df	p
**Hazard Ratio and Proportionality**	0.877	(0.798, 0.964)	4.968	1	0.026

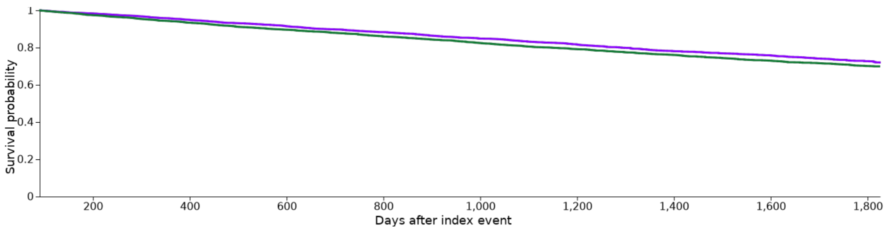

2,241 patients in Cohort 1 and 2,258 patients in Cohort 2 were excluded from results because they had the outcome prior to the time window.

Number of instances excluding patients with outcome prior to the time window

Cohort	Patients in cohort	Patients with outcome	Mean	Standard	Median

					Deviation	
1	(new)DM+AKDwithGLP-1	5,251	777	4.103	7.560	2
2	(new)DM+AKDwithoutGLP-1	5,234	982	2.650	4.763	1

	t	df	p
**Test Statistics**	4.916	1757	0.000

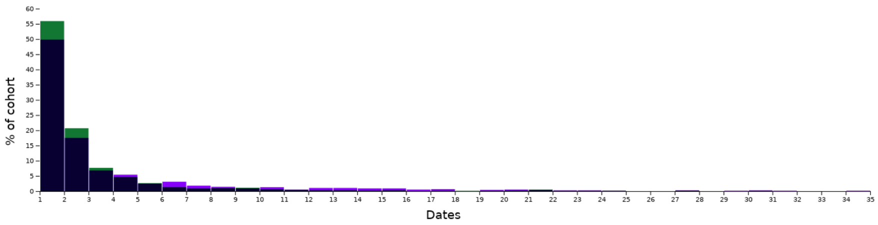

9 data points for Cohort 1 and 3 data points for Cohort 2 were omitted for display purposes.

2,241 patients in Cohort 1 and 2,258 patients in Cohort 2 were excluded from results because they had the outcome prior to the time window.

Suppl. Table 6. Risk of MAKE in type 2 diabetes patients with AKD: comparison between GLP-1 RAs users and non-users after propensity score matching. The results presented in the table below are from an analysis of a cohort of patients after propensity score matching. The baseline cohort included patients with a history of MAKE, who were excluded from the analysis. Statistical analysis was performed using the two-sided log-rank test and risk analysis, with no adjustments for multiple comparisons. The table provides detailed statistical results, including test statistics (e.g., z, χ^2), confidence intervals, effect sizes (hazard ratios, risk differences, risk ratios, odds ratios), degrees of freedom, exact P values and 95% confidence intervals.

MAKE

Risk analysis excluding patients with outcome prior to the time window

Cohort	Patients in cohort	Patients with outcome	Risk
1 (new)DM+AKDwithGLP-1	6,513	701	0.108
2 (new)DM+AKDwithoutGLP-1	6,478	1,034	0.160

		95% CI	z	p
**Risk Difference**	-0.052	(-0.064, -0.040)	-8.709	0.000
**Risk Ratio**	0.674	(0.617, 0.737)	N/A	N/A
**Odds Ratio**	0.635	(0.573, 0.704)	N/A	N/A

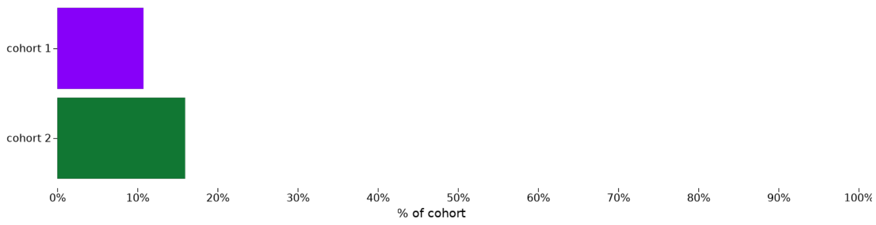

979 patients in Cohort 1 and 1,014 patients in Cohort 2 were excluded from results

because they had the outcome prior to the time window.

Kaplan - Meier survival analysis excluding patients with outcome prior to the time window

Cohort	Patients in cohort	Patients with outcome	Median survival (days)	Survival probability at end of time window
1 (new)DM+AKDwithGLP-1	6,513	701	--	79.45%
2 (new)DM+AKDwithoutGLP-1	6,478	1,034	--	73.64%

	χ^2	df	p
**Log-Rank Test**	42.437	1	0.000

	Hazard Ratio	95% CI	χ^2	df	p
**Hazard Ratio and Proportionality**	0.728	(0.661, 0.801)	2.111	1	0.146

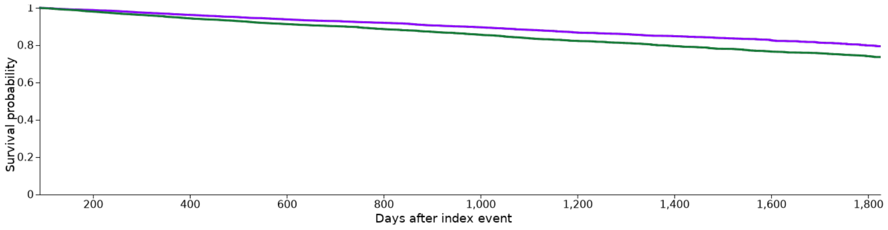

979 patients in Cohort 1 and 1,014 patients in Cohort 2 were excluded from results because they had the outcome prior to the time window.

Number of instances excluding patients with outcome prior to the time window

Cohort	Patients in cohort	Patients with outcome	Mean	Standard Deviati	Median

					on	
1	(new)DM+AKDwithGLP-1	6,513	701	3.310	5.613	1
2	(new)DM+AKDwithoutGLP-1	6,478	1,034	2.945	6.662	1

	t	df	p
**Test Statistics**	1.191	1733	0.234

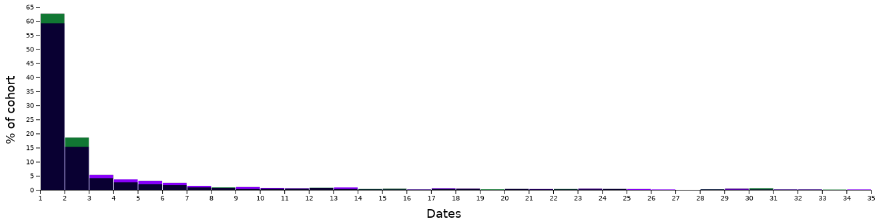

4 data points for Cohort 1 and 3 data points for Cohort 2 were omitted for display purposes.

979 patients in Cohort 1 and 1,014 patients in Cohort 2 were excluded from results because they had the outcome prior to the time window.

Suppl. Table 7. Sensitivity analysis for all-cause mortality, MACE, and MAKE between GLP-1 RAs users and non-users

	Mortality aHR (95%CI)	MACE aHR (95%CI)	MAKE aHR (95%CI)
**All eligible subjects without weighting (n=174,107)**	0.44 (0.40-0.48)		
1:1 PSM, caliper=0.2	0.57 (0.51-0.64)	0.73 (0.66-0.80)	0.88 (0.80-0.96)
**Eligible subjects with different enrolled period, 180-day mortality, 1:1 PSM**			
Patient enrolled in after 2006	0.62 (0.56-0.68)	0.78 (0.71-0.84)	0.90 (0.82-0.98)
Patient enrolled in before 2007	0.31 (0.03-2.97)	0.22 (0.03-1.86)	0.42 (0.09-2.10)
Patient enrolled in before 2008	0.31 (0.06-1.48)	0.47 (0.13-1.79)	0.87 (0.27-2.73)
Patient enrolled in before 2015	0.41 (0.30-0.57)	0.34 (0.21-0.53)	0.54 (0.35-0.82)
Patient enrolled in before 2020	0.59 (0.52-0.68)	0.59 (0.50-0.71)	0.73 (0.61-0.87)
Patients enrolled between 2020 and 2022	0.70 (0.59-0.84)	0.86 (0.71-1.04)	0.96 (0.79-1.16)
**Eligible subjects with different outcome definition, 1:1 PSM**			
Include died within 3 months after discharge	0.57 (0.53-0.62)	0.64 (0.57-0.73)	0.81 (0.71-0.92)
**Cox regression models with different covariates**			
Model 1 (age and gender, ethnicity)	0.52 (0.47-0.58)	0.63 (0.56-0.72)	0.79 (0.69-0.89)
Model 2 (age, gender, ethnicity, co-morbidities)	0.53 (0.48-0.59)	0.61 (0.54-0.68)	0.79 (0.69-0.87)
Model 3 (final full model except AKI etiology)	0.57 (0.51-0.63)	0.66 (0.59-0.74)	0.78 (0.69-0.87)
Model 4 (final full model including AKI etiology)	0.59 (0.54-0.64)	0.69 (0.63-0.77)	0.82 (0.74-0.91)

Abbreviations: aHR, adjusted hazzard ratio; CI, confidence interval; GLP-1 RAs; glucagon-like peptide-1 receptor agonists; MACE, major adverse cardiac events; MAKE, major adverse kidney events; PSM, propensity score matching

Suppl. Table 8. Sensitivity analysis for incidence of outcomes of interest among GLP-1 RAs users compared to non-users after propensity score matching in patients with eGFR ≥ 30 ml/min/1.73m^2 treated with Exenatide

Outcome	Patients with outcome		aHR (95%CI)
	GLP-1 RAs group	Control group	
**Primary outcome**			
Mortality	16.1% (74/461)	14.5% (67/461)	0.93 (0.67-1.30)
**Secondary outcome**			
MACE	18.7% (57/305)	19.9% (58/292)	0.77 (0.54-1.12)
MAKE	19.3% (75/388)	18.1% (70/387)	0.90 (0.65-1.24)

Abbreviations: aHR, adjusted hazard ratio; MACE, major adverse cardiac events; MAKE, major adverse kidney events; GLP-1 RAs; glucagon-like peptide 1 receptor agonists

Suppl. Table 9. Sensitivity analysis for incidence of outcomes of interest among GLP-1 RAs users compared to non-users after propensity score matching in patients with eGFR < 30 ml/min/1.73m^2 treated with Exenatide

Outcome	Patients with outcome		aHR (95%CI)
	GLP-1 RAs group	Control group	
**Primary outcome**			
Mortality	20.3% (16/79)	26.6% (21/79)	0.65 (0.34-1.25)
**Secondary outcome**			
MACE	28.6% (12/42)	29.1% (16/55)	0.76 (0.40-1.81)
MAKE	34.0% (18/53)	33.3% (14/42)	0.78 (0.39-1.58)

Abbreviations: aHR, adjusted hazard ratio; MACE, major adverse cardiac events; MAKE, major adverse kidney events; GLP-1 RA*s;* glucagon-like peptide 1 receptor agonists

Suppl. Table 10. Sensitivity analysis for all-cause mortality, MACE, and MAKE between GLP-1 RAs users and other second-line antihyperglycemic treatments users (Sulfonylureas, dipeptidyl peptidase-4 inhibitor or Pioglitazone) user in a new-user design.

Outcome	Patients with outcome		aHR (95%CI)
	GLP-1 RAs group	Other active treatment group	
**Primary outcome**			
Mortality	7.5% (313/4164)	16.5% (689/4164)	0.49 (0.41-0.58)
**Secondary outcome**			
MACE	16.3% (161/989)	29.6% (528/1785)	0.63 (0.55-0.71)
MAKE	9.3% (387/4164)	21.9% (910/4164)	0.72 (0.61-0.83)

Abbreviations: aHR, adjusted hazard ratios; CI, confidence interval; GLP-1 RAs; glucagon-like peptide-1 receptor agonists; MACE, major adverse cardiac events; MAKE, major adverse kidney events

(E) Supplementary figures

Suppl. Figure 1. Comparative HbA1C mean levels at baseline, D0-30, and D60-90 for GLP-1 RAs users and the control group. The graph shows the mean HbA1C levels at baseline, days 0-30, and days 60-90 for the GLP-1 RAs users (blue line) and the control group (red line). Data are presented as mean values with error bars indicating the standard deviation. An asterisk (*) indicates statistically significant differences (P < 0.05) between the two groups at each time point.

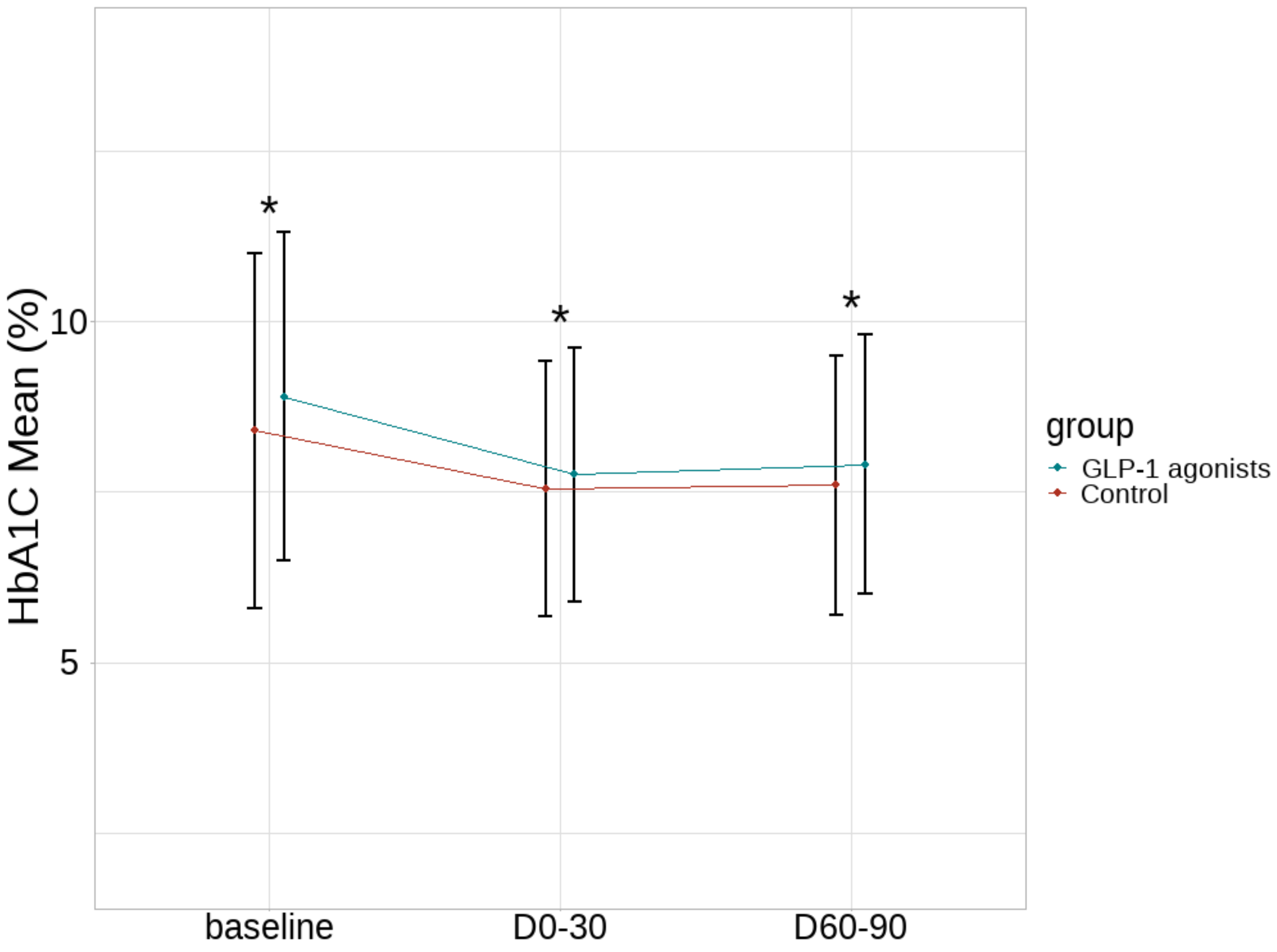

Abbreviations: GLP-1 RAs; glucagon-like peptide-1 receptor agonists; HbA1C, glycated hemoglobin

Suppl. Figure 2. Comparative eGFR mean levels at baseline, D0-30, and D60-90 for GLP-1 RAs users and the control group. The graph shows the mean eGFR levels at baseline, days 0-30, and days 60-90 for the GLP-1 RAs users (blue line) and the control group (red line). Data are presented as mean values with error bars indicating the standard deviation. An asterisk (*) indicates statistically significant differences (P < 0.05) between the two groups at each time point.

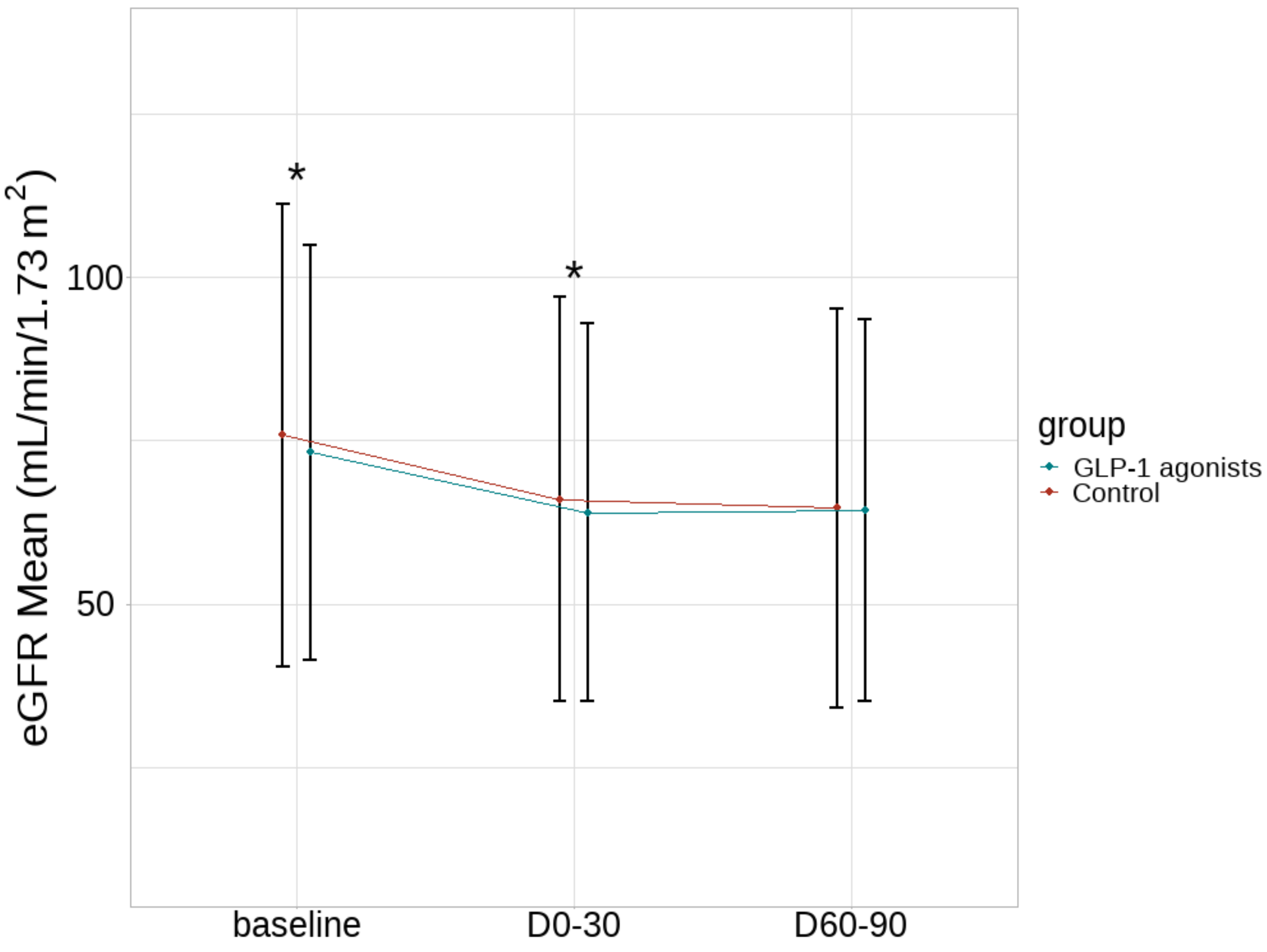

Abbreviations: eGFR, estimated glomerular filtration rate; GLP-1 RAs; glucagon-like peptide-1 receptor agonists.

Suppl. Figure 3. Comparative HbA1C mean levels at baseline, D0-30, and D60-90 for GLP-1 RAs users and other second-line antihyperglycemic treatments users. The graph shows the mean HbA1c levels at baseline, days 0-30, and days 60-90 for the GLP-1 RAs users (blue line) and other second-line antihyperglycemic treatments users (red line). Data are presented as mean values with error bars indicating the standard deviation. An asterisk (*) indicates statistically significant differences ($P < 0.05$) between the two groups at each time point.

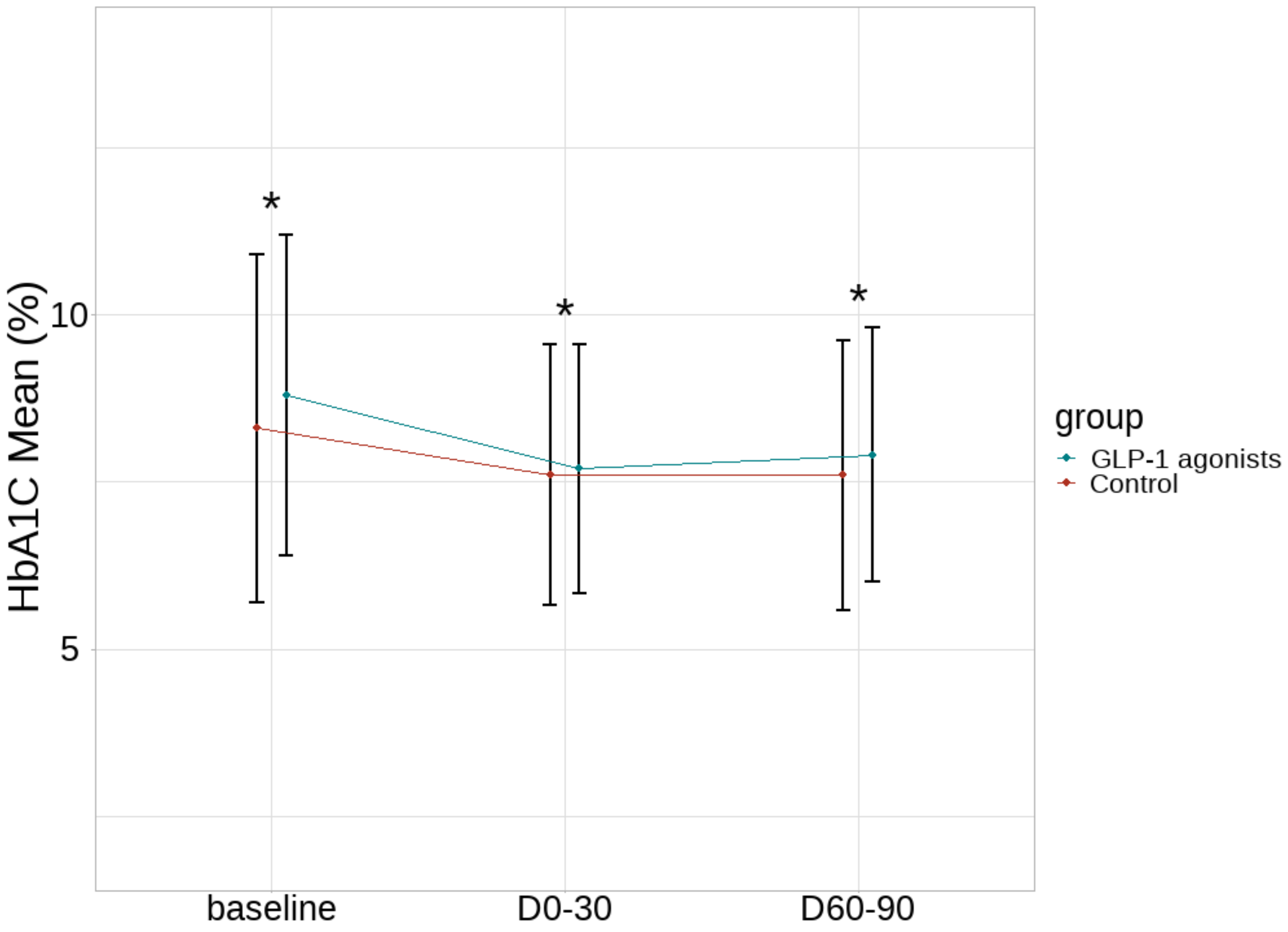

Abbreviations: GLP-1 RAs; glucagon-like peptide-1 receptor agonists; HbA1C, glycated hemoglobin

Suppl. Figure 4. Positive outcome control, negative outcome control, and specificity analysis
Forest plots of adjusted hazard ratios (aHRs) for the GLP-1 RAs users (n=7492) versus non-users (n=7492) during the AKD period regarding positive control, negative outcome controls, and specific analysis. The HRs were adjusted for age, sex, and race due to their potential interactions with kidney disease. AHRs (center) and 95% CIs (error bars) are presented. The vertical line indicates an aHR of 1.00; lower limits of 95% CIs with values greater than 1.00 indicate a significantly increased risk. Independent samples were used, with each sample derived from different subjects. Data collection involved independent measurements from each patient. Control groups are defined as non-users of GLP-1 RAs.

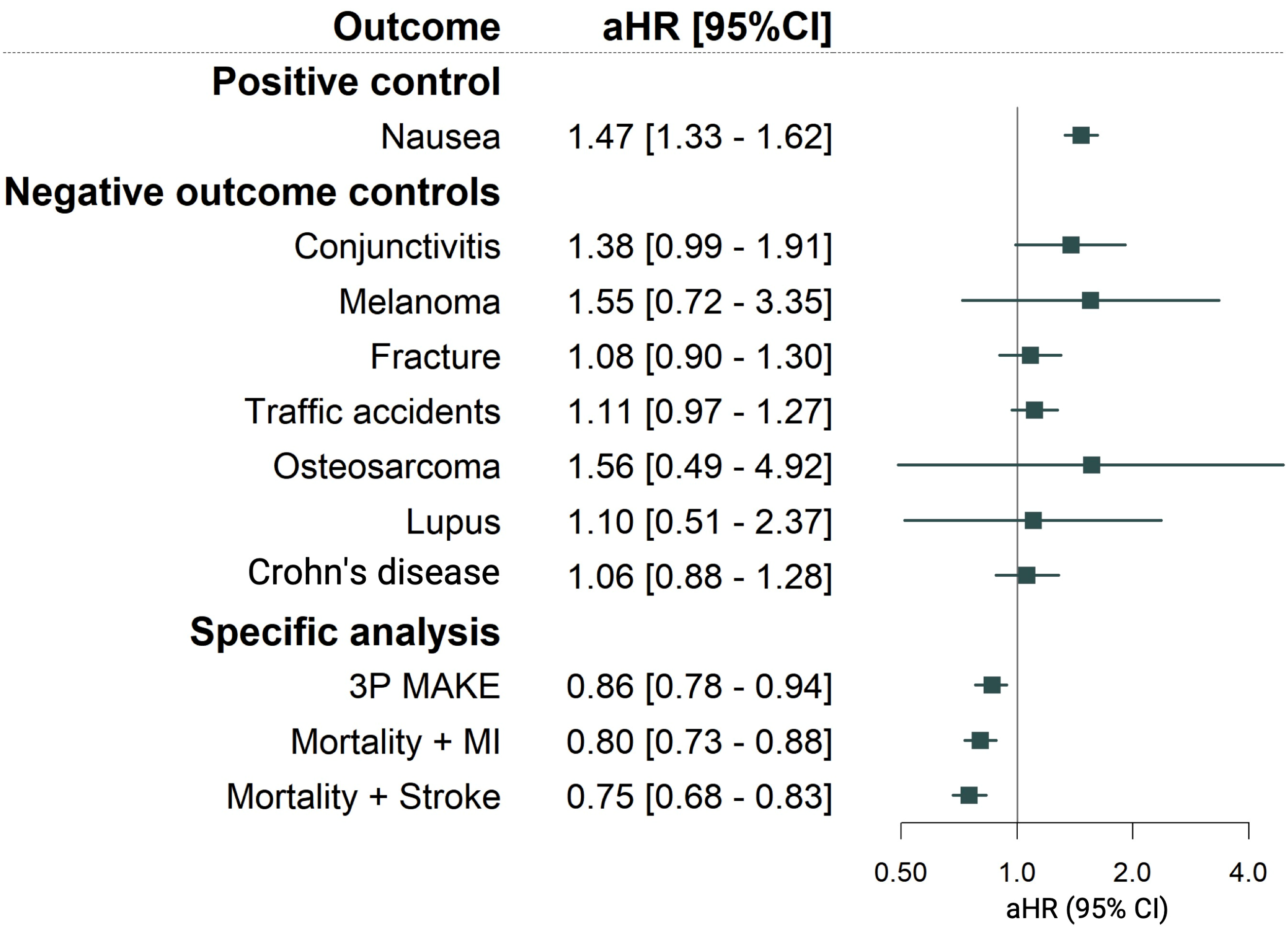

Abbreviations: aHR, adjusted hazard ratio; CI, confidence interval; DPP4i, ESRD, end-stage renal disease; HF, heart failure; MI, myocardial infarction; inhibitor; 3P MAKE, 3 points major adverse kidney event

* 3P MAKE: redialysis, dialysis dependence, or eGFR < 15 ml/min/1.73 m^2

Suppl. Figure 5. Graphic abstract for the primary study

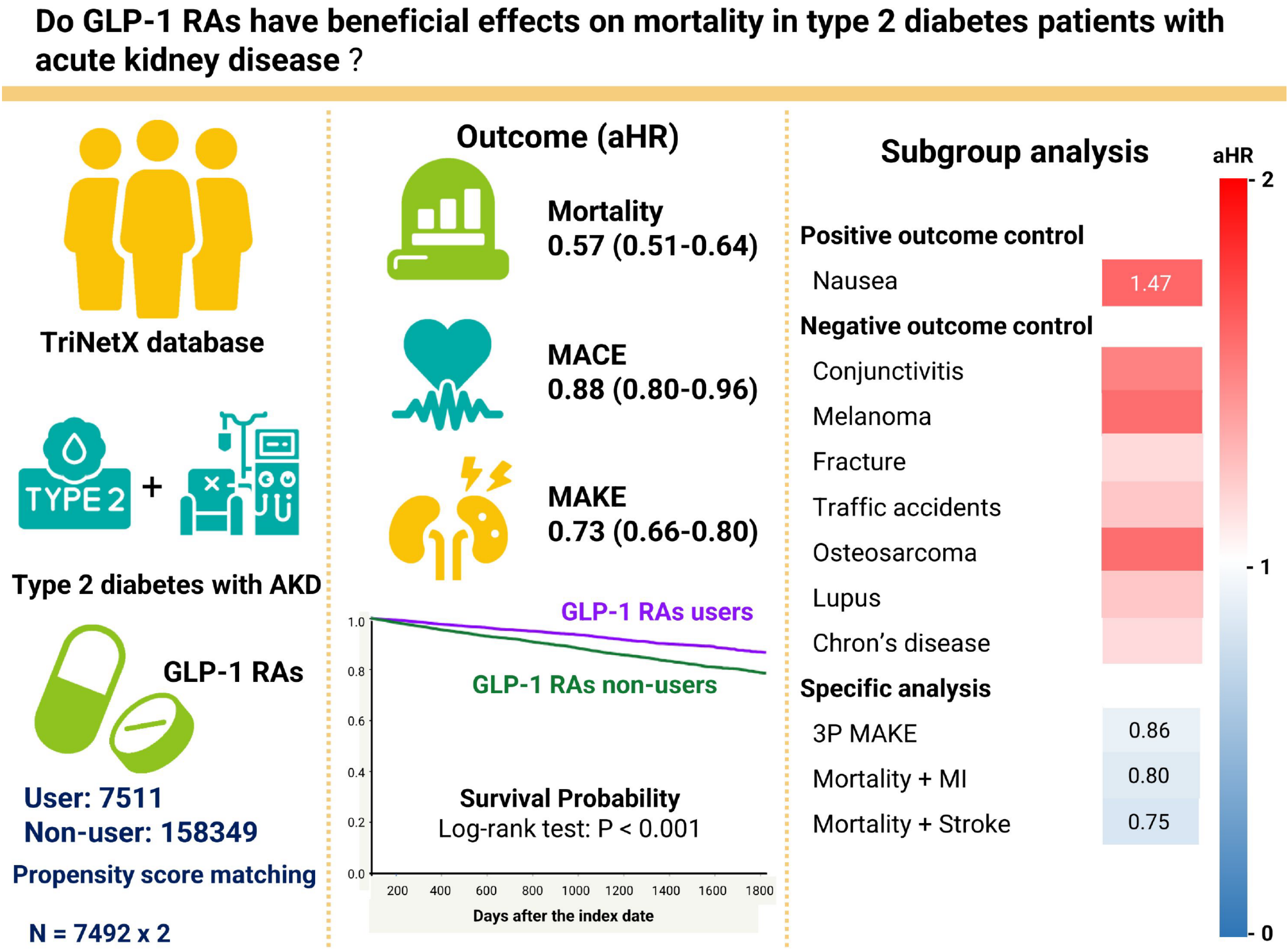

Abbreviations: aHR, adjusted hazard ratio; AKD, acute kidney disease; GLP-1 RAs, glucagon-like peptide-1 receptor agonists; MACE, major adverse cardiac event; MAKE, major adverse kidney event; MI, myocardial infarction

Suppl. Figure 6. External validation by CGRD database. The Kaplan-Meier curves presented the long-term outcomes of interest, including (A) MACE (B) MAKE. The blue line corresponds to GLP-1 RAs users, and the yellow line represents GLP-1 RAs non-users. The number at risk at different time points is shown below the curves.

(A)

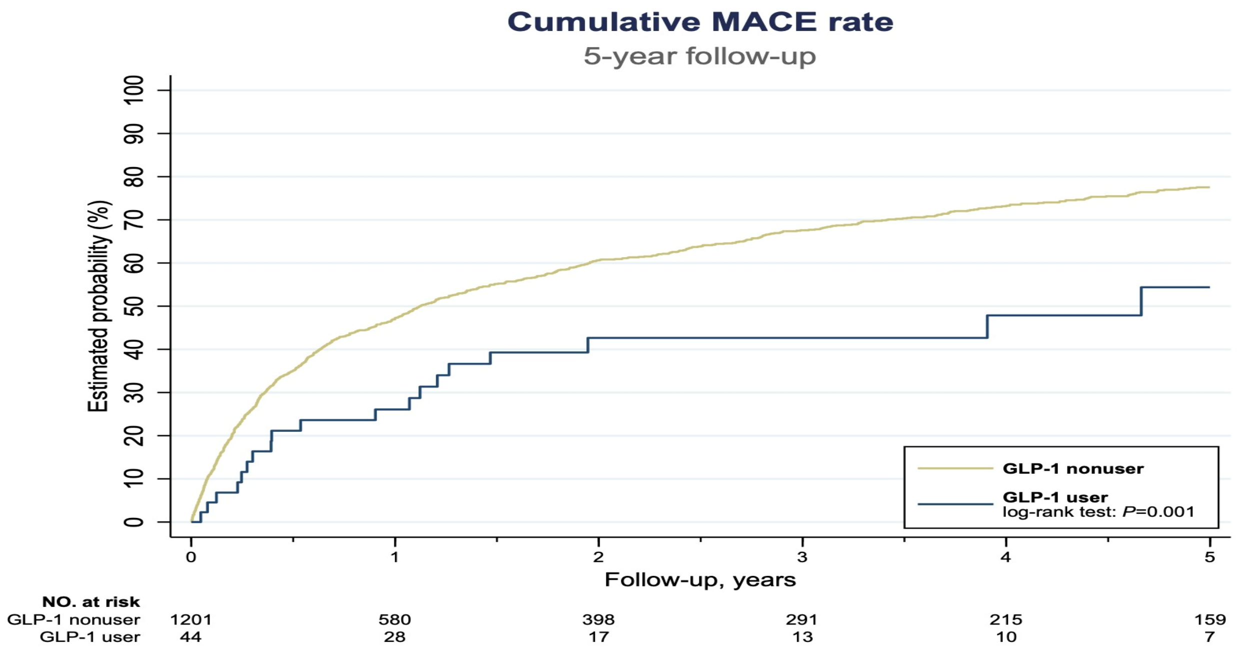

(B)

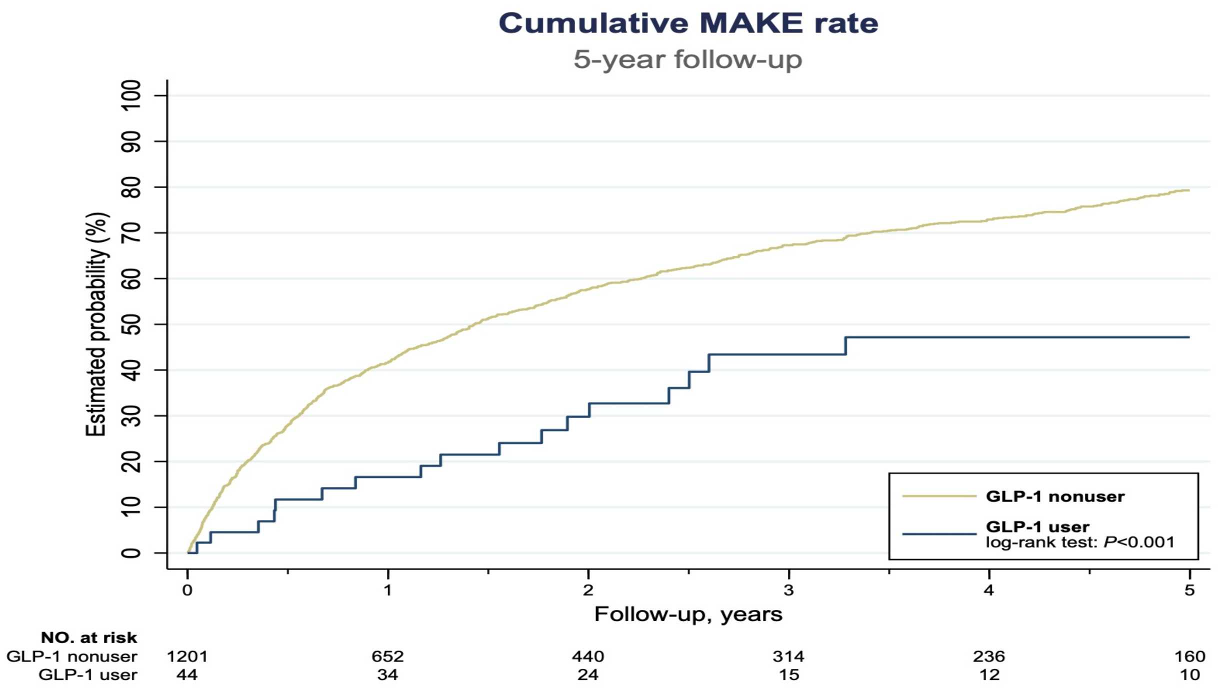

Abbreviations: CGRD, Chang Gung Research Database; GLP-1 RAs, glucagon-like peptide-1 receptor agonists; MACE, major adverse cardiac event; MAKE, major adverse kidney event.

(F) Supplementary reference

1. Topaloglu, U. & Palchuk, M.B. Using a Federated Network of Real-World Data to Optimize Clinical Trials Operations. *JCO Clin Cancer Inform* **2**, 1-10 (2018).
2. MacKenzie, S.L., Wyatt, M.C., Schuff, R., Tenenbaum, J.D. & Anderson, N. Practices and perspectives on building integrated data repositories: results from a 2010 CTSA survey. *J Am Med Inform Assoc* **19**, e119-124 (2012).
3. Hudson, C.L., Topaloglu, U., Bian, J., Hogan, W. & Kieber-Emmons, T. Automated Tools for Clinical Research Data Quality Control using NCI Common Data Elements. *AMIA Jt Summits Transl Sci Proc* **2014**, 60-69 (2014).
4. Kahn, M.G.*, et al.* Transparent reporting of data quality in distributed data networks. *EGEMS (Wash DC)* **3**, 1052 (2015).
5. Weiskopf, N.G., Hripcsak, G., Swaminathan, S. & Weng, C. Defining and measuring completeness of electronic health records for secondary use. *J Biomed Inform* **46**, 830-836 (2013).
6. Shao, S.C.*, et al.* The Chang Gung Research Database—a multi-institutional electronic medical records database for real-world epidemiological studies in Taiwan. *Pharmacoepidemiology and drug safety* **28**, 593-600 (2019).
7. Hsiao, F.-C., Lin, C.-P., Tung, Y.-C., Wu, C.-T. & Chu, P.-H. Major adverse limb events in type 2 diabetes patients receiving glucagon-like peptide-1 receptor agonists versus sodium-glucose cotransporter 2 inhibitors: a retrospective multi-institutional study. *Diabetes Research and Clinical Practice* **180**, 109076 (2021).
8. Tsai, C.J. & Tsao, C.F. Comparison of Glucose Lowering Efficacy of Human GLP-1 Agonist in Taiwan Type 2 Diabetes Patients after Switching from DPP-4 Inhibitor Use or Non-Use. *J Pers Med* **12**(2022).
9. Su, Y.-C.*, et al.* Comparison of sodium-glucose cotransporter 2 inhibitors vs glucagonlike peptide-1 receptor agonists and incidence of dry eye disease in patients with type 2 diabetes in Taiwan. *JAMA network open* **5**, e2232584-e2232584 (2022).
10. Lin, T.Y.*, et al.* Risk of Diabetic Retinopathy between Sodium-Glucose Cotransporter-2 Inhibitors and Glucagon-Like Peptide-1 Receptor Agonists. *Diabetes Metab J* **47**, 394-404 (2023).
11. Lin, Y.*, et al.* The cardiovascular and renal effects of glucagon-like peptide 1 receptor agonists in patients with advanced diabetic kidney disease. *Cardiovasc Diabetol* **22**, 60 (2023).
12. Tsai, M.-S.*, et al.* Chang Gung Research Database: A multi-institutional database consisting of original medical records. *biomedical journal* **40**, 263-269 (2017).

13. Sheahan, K.H., Wahlberg, E.A. & Gilbert, M.P. An overview of GLP-1 agonists and recent cardiovascular outcomes trials. *Postgrad Med J* **96**, 156-161 (2020).
14. Iepsen, E.W., Torekov, S.S. & Holst, J.J. Liraglutide for Type 2 diabetes and obesity: a 2015 update. *Expert Rev Cardiovasc Ther* **13**, 753-767 (2015).
15. Filippatos, T.D. & Elisaf, M.S. Effects of glucagon-like peptide-1 receptor agonists on renal function. *World J Diabetes* **4**, 190-201 (2013).
16. Muskiet, M.H.A.*, et al.* GLP-1 and the kidney: from physiology to pharmacology and outcomes in diabetes. *Nat Rev Nephrol* **13**, 605-628 (2017).
17. Steveling, E.H., Winzeler, B. & Bircher, A.J. Systemic Allergic Reaction to the GLP-1 Receptor Agonist Exenatide. *J Pharm Technol* **30**, 182-186 (2014).
18. Smith, D.K. & Wessner, M.J. Cochrane for Clinicians: DPP-4 Inhibitors and GLP-1 Receptor Agonists for Prevention or Delay of Type 2 Diabetes Mellitus and Associated Complications. *American Family Physician* **97**, 437 (2018).
19. Pan, H.-C.*, et al.* Sodium-Glucose Cotransport Protein 2 Inhibitors in Patients With Type 2 Diabetes and Acute Kidney Disease. *JAMA Network Open* **7**, e2350050-e2350050 (2024).
20. Marso, S.P.*, et al.* Semaglutide and cardiovascular outcomes in patients with type 2 diabetes. *New England Journal of Medicine* **375**, 1834-1844 (2016).
21. Marso, S.P.*, et al.* Liraglutide and cardiovascular outcomes in type 2 diabetes. *New England Journal of Medicine* **375**, 311-322 (2016).
22. Holman, R.R.*, et al.* Effects of once-weekly exenatide on cardiovascular outcomes in type 2 diabetes. *New England Journal of Medicine* **377**, 1228-1239 (2017).

(G) STROBE statement checklist

STROBE Statement—Checklist of items that should be included in reports of ***cohort studies***

	Item No	Recommendation	Page No
**Title and abstract**	1	(*a*) Indicate the study's design with a commonly used term in the title or the abstract	P.1, P.3
		(*b*) Provide in the abstract an informative and balanced summary of what was done and what was found	P.3
**Introduction**			
Background/rationale	2	Explain the scientific background and rationale for the investigation being reported	P.4-5
Objectives	3	State specific objectives, including any prespecified hypotheses	P.5
**Methods**			
Study design	4	Present key elements of study design early in the paper	P.16-17
Setting	5	Describe the setting, locations, and relevant dates, including periods of recruitment, exposure, follow-up, and data collection	P.14-15
Participants	6	(*a*) Give the eligibility criteria, and the sources and methods of selection of participants. Describe methods of follow-up	P.16-17
		(*b*) For matched studies, give matching criteria and number of exposed and unexposed	P.17
Variables	7	Clearly define all outcomes, exposures, predictors, potential confounders, and effect modifiers. Give diagnostic criteria, if applicable	P.15-16, Suppl. P.11-13
Data sources/ measurement	8*	For each variable of interest, give sources of data and details of methods of assessment (measurement). Describe comparability of assessment methods if there is more than one group	P.15, Suppl. P.3-10
Bias	9	Describe any efforts to address potential sources of bias	P.18-19
Study size	10	Explain how the study size was arrived at	P.16-17
Quantitative variables	11	Explain how quantitative variables were handled in the analyses. If applicable, describe which groupings were chosen and why	P.16
Statistical methods	12	(*a*) Describe all statistical methods, including those used to control for confounding	P.18-19
		(*b*) Describe any methods used to examine subgroups and interactions	P.17-18
		(*c*) Explain how missing data were addressed	P.18
		(*d*) If applicable, explain how loss to follow-up was addressed	P.18
		(*e*) Describe any sensitivity analyses	P.19, Suppl P.9
**Results**			
Participants	13*	(a) Report numbers of individuals at each stage of study—eg numbers potentially eligible, examined for eligibility, confirmed eligible, included in the study, completing follow-up, and analysed	P.5-6
		(b) Give reasons for non-participation at each stage	P.5-6
		(c) Consider use of a flow diagram	Figure 1
Descriptive data	14*	(a) Give characteristics of study participants (eg demographic, clinical, social) and information on exposures and potential confounders	P.5-6, Table 1, Table S1-3, Figure S1-2
		(b) Indicate number of participants with missing data for each variable of interest	P.5-6
		(c) Summarise follow-up time (eg, average and total amount)	P.5-6
Outcome data	15*	Report numbers of outcome events or summary measures over time	P.6-7

Main results	16	(*a*) Give unadjusted estimates and, if applicable, confounder-adjusted estimates and their precision (eg, 95% confidence interval). Make clear which confounders were adjusted for and why they were included<br>(*b*) Report category boundaries when continuous variables were categorized<br>(*c*) If relevant, consider translating estimates of relative risk into absolute risk for a meaningful time period	P.6-7, Table 2<br>N/A<br>P.6-7, Table S4-6
Other analyses	17	Report other analyses done—eg analyses of subgroups and interactions, and sensitivity analyses	P.7-9, Figure 3, Table S7-10, Figure S3-6
**Discussion**			
Key results	18	Summarise key results with reference to study objectives	P.9
Limitations	19	Discuss limitations of the study, taking into account sources of potential bias or imprecision. Discuss both direction and magnitude of any potential bias	P.13-14
Interpretation	20	Give a cautious overall interpretation of results considering objectives, limitations, multiplicity of analyses, results from similar studies, and other relevant evidence	P.14
Generalisability	21	Discuss the generalisability (external validity) of the study results	P.14
**Other information**			
Funding	22	Give the source of funding and the role of the funders for the present study and, if applicable, for the original study on which the present article is based	P.24

*Give information separately for exposed and unexposed groups.

Note: An Explanation and Elaboration article discusses each checklist item and gives methodological background and published examples of transparent reporting. The STROBE checklist is best used in conjunction with this article (freely available on the Web sites of PLoS Medicine at http://www.plosmedicine.org/, Annals of Internal Medicine at http://www.annals.org/, and Epidemiology at http://www.epidem.com/). Information on the STROBE Initiative is available at http://www.strobe-statement.org.
