# Supplementary material for: GLP-1 receptor agonists’ impact on cardio-renal outcomes and mortality in T2D with acute kidney disease
Source: Nat Commun. 2024 Jul 13;15:5912. doi: 10.1038/s41467-024-50199-y (PMC11246471; doi:10.1038/s41467-024-50199-y)
Supplement: Supplementary file 3 — Reporting Summary [file 41467_2024_50199_MOESM3_ESM.pdf]

nature portfolio

Corresponding author(s): Vin-Cent Wu

Last updated by author(s): Apr 25, 2024

Reporting Summary

Nature Portfolio wishes to improve the reproducibility of the work that we publish. This form provides structure for consistency and transparency in reporting. For further information on Nature Portfolio policies, see our Editorial Policies and the Editorial Policy Checklist.

Statistics

For all statistical analyses, confirm that the following items are present in the figure legend, table legend, main text, or Methods section.

n/a	Confirmed	
☐	☒	The exact sample size (n) for each experimental group/condition, given as a discrete number and unit of measurement
☒	☐	A statement on whether measurements were taken from distinct samples or whether the same sample was measured repeatedly
☐	☒	The statistical test(s) used AND whether they are one- or two-sided<br>*Only common tests should be described solely by name; describe more complex techniques in the Methods section.*
☐	☒	A description of all covariates tested
☐	☒	A description of any assumptions or corrections, such as tests of normality and adjustment for multiple comparisons
☐	☒	A full description of the statistical parameters including central tendency (e.g. means) or other basic estimates (e.g. regression coefficient) AND variation (e.g. standard deviation) or associated estimates of uncertainty (e.g. confidence intervals)
☐	☒	For null hypothesis testing, the test statistic (e.g. F, t, r) with confidence intervals, effect sizes, degrees of freedom and P value noted<br>*Give P values as exact values whenever suitable.*
☒	☐	For Bayesian analysis, information on the choice of priors and Markov chain Monte Carlo settings
☒	☐	For hierarchical and complex designs, identification of the appropriate level for tests and full reporting of outcomes
☒	☐	Estimates of effect sizes (e.g. Cohen's d, Pearson's r), indicating how they were calculated

Our web collection on statistics for biologists contains articles on many of the points above.

Software and code

Policy information about availability of computer code

Data collection	Data for this investigation were accessed through the TriNetX Analytics platform, a global collaborative health research network widely used in numerous prominent epidemiological studies (Supplementary appendix)19-21. The dataset used in this study was extensive, encompassing various aspects of patient information including demographic details, diagnoses (according to International Classification of Diseases, Tenth Revision, Clinical Modification [ICD-10-CM] codes), procedures (classified according to the International Classification of Diseases, Tenth Revision, Procedure Coding System [ICD-10-PCS] or Current Procedural Terminology), and medications (coded as per the Veterans Affairs National Formulary). It also covered laboratory tests (organized using Logical Observation Identifiers Names and Codes) and healthcare utilization records from a network of 79 healthcare organizations including hospitals, primary care facilities, and specialist care providers. The extensive dataset incorporated data from both insured and uninsured patients, comprising a participant pool exceeding 250 million individuals. The dataset spans a time period from September 1, 2002, to December 1, 2022.
Data analysis	We used R software (version 3.2.2, Free Software Foundation, Inc, Boston, MA), SAS (version 9.2, SAS Inc., Cary, NC), and Stata/MP (version 16, StataCorp, College Station, TX).

For manuscripts utilizing custom algorithms or software that are central to the research but not yet described in published literature, software must be made available to editors and reviewers. We strongly encourage code deposition in a community repository (e.g. GitHub). See the Nature Portfolio guidelines for submitting code & software for further information.

Data

Policy information about availability of data

All manuscripts must include a data availability statement. This statement should provide the following information, where applicable:

- Accession codes, unique identifiers, or web links for publicly available datasets
- A description of any restrictions on data availability
- For clinical datasets or third party data, please ensure that the statement adheres to our policy

The datasets used and/or analyzed during the current study are available from the corresponding author on reasonable request.

Research involving human participants, their data, or biological material

Policy information about studies with human participants or human data. See also policy information about sex, gender (identity/presentation), and sexual orientation and race, ethnicity and racism.

Reporting on sex and gender	In our study, we have reported exclusively on 'sex' as we investigated biological factors. The necessary distribution of study participants or samples has been presented in the 'Results' section of our report.
Reporting on race, ethnicity, or other socially relevant groupings	In this study, the racial and ethnic categories represented were 'White' and 'Not Hispanic or Latino'. These data were provided by healthcare organizations (HCOs) through the TriNetX database. We included this demographic information in our analysis to explore potential variations in the effects of glucagon-like peptide-1 receptor agonists across these groups, thereby enhancing the robustness and generalizability of our findings.
Population characteristics	In the cohort consisting of 165,860 AKD (acute kidney disease) patients eligible for withdrawal from acute dialysis, the average age was 59.0 years. The gender distribution was nearly balanced with 49.7% male and 50.3% female. Regarding race and ethnicity, 65.1% of the patients were identified as White, and 79.8% were classified as Not Hispanic or Latino.
Recruitment	The inclusion criteria were age 18 to 90 years, a confirmed diabetes diagnosis, and ever dialysis during their hospital stay.
Ethics oversight	Data analysis utilizing the TriNetX platform received approval from the Institutional Review Board of Chi-Mei Hospital (No: 11202-002), as well as approval from the institutional review boards of all participating hospitals.

Note that full information on the approval of the study protocol must also be provided in the manuscript.

Field-specific reporting

Please select the one below that is the best fit for your research. If you are not sure, read the appropriate sections before making your selection.

☒ Life sciences ☐ Behavioural & social sciences ☐ Ecological, evolutionary & environmental sciences

For a reference copy of the document with all sections, see nature.com/documents/nr-reporting-summary-flat.pdf

Life sciences study design

All studies must disclose on these points even when the disclosure is negative.

Sample size	In this retrospective observational cohort study, the sample size was determined based on the statistical power needed to detect significant effects of glucagon-like peptide-1 receptor agonists on cardio-renal outcomes and mortality among patients with type 2 diabetes experiencing acute kidney disease. The database provided a comprehensive dataset covering a wide demographic, ensuring robust representation of the target population. Sample size calculations were performed using standard power analysis techniques, aiming for a power of 90% and an alpha of 0.05, to ensure sufficient sensitivity to detect clinically relevant differences. Preliminary estimates of effect sizes were derived from existing literature on similar interventions. Potential biases such as selection bias and information bias were addressed through rigorous data curation and the application of advanced statistical adjustments, including multivariable regression models, to account for confounding variables. This approach ensured that the sample size was adequate to provide reliable and valid conclusions.
Data exclusions	The patients who had an eGFR < 15 ml/min/1.73m2 before the index hospitalization and either remained on dialysis, required re-dialysis, or died within 3 months post-discharge were excluded.
Replication	We have external validation by a multicenter dataset.
Randomization	This is a retrospective observation cohort study, so we did not have any design about randomization.
Blinding	This is a retrospective observation cohort study, so we did not have any design about blinding.

Reporting for specific materials, systems and methods

We require information from authors about some types of materials, experimental systems and methods used in many studies. Here, indicate whether each material, system or method listed is relevant to your study. If you are not sure if a list item applies to your research, read the appropriate section before selecting a response.

Materials & experimental systems

n/a	Involved in the study
☒	☐ Antibodies
☒	☐ Eukaryotic cell lines
☒	☐ Palaeontology and archaeology
☒	☐ Animals and other organisms
☐	☒ Clinical data
☒	☐ Dual use research of concern
☒	☐ Plants

Methods

n/a	Involved in the study
☒	☐ ChIP-seq
☒	☐ Flow cytometry
☒	☐ MRI-based neuroimaging

Clinical data

Policy information about clinical studies
All manuscripts should comply with the ICMJEguidelines for publication of clinical research and a completedCONSORT checklist must be included with all submissions.

Clinical trial registration	This is a retrospective observation cohort study, so we do not have registration number.
Study protocol	We identified 165,860 patients diagnosed with type 2 diabetes and AKD upon admission to the enrolled healthcare facilities during study period. Patients with AKD were defined as those who were discharged and able to wean from acute kidney injury (AKI) requiring dialysis. For all participates, the index date was determined as the 90 days following their hospital discharge. The inclusion criteria were age 18 to 90 years, a confirmed diabetes diagnosis, and ever dialysis during their hospital stay. The patients who had an eGFR < 15 ml/min/1.73m2 before the index hospitalization and either remained on dialysis, required re-dialysis, or died within 3 months post-discharge were excluded. We categorized patients as GLP-1 RAs users if they had been prescribed a GLP-1 RAs at AKD. The cohort was divided into two groups: the GLP-1 RAs users group (n=7,511), and the GLP-1 RAs non-users group (n=158,349). Propensity score matching (PSM) was performed using 25 variables detailed in the "Covariates" section. All patients were closely tracked for up to 5 years for any occurrence of the outcome of interests. To counteract potential protopathic or ascertainment bias, any instances of primary and secondary outcomes that manifested before the designated index date were disregarded, prompting a repeat of the PSM process.
Data collection	Data for this investigation were accessed through the TriNetX Analytics platform, a global collaborative health research network widely used in numerous prominent epidemiological studies (Supplementary appendix)19-21. The dataset used in this study was extensive, encompassing various aspects of patient information including demographic details, diagnoses (according to International Classification of Diseases, Tenth Revision, Clinical Modification [ICD-10-CM] codes), procedures (classified according to the International Classification of Diseases, Tenth Revision, Procedure Coding System [ICD-10-PCS] or Current Procedural Terminology), and medications (coded as per the Veterans Affairs National Formulary). It also covered laboratory tests (organized using Logical Observation Identifiers Names and Codes) and healthcare utilization records from a network of 79 healthcare organizations including hospitals, primary care facilities, and specialist care providers. The extensive dataset incorporated data from both insured and uninsured patients, comprising a participant pool exceeding 250 million individuals. The dataset spans a time period from September 1, 2002, to December 1, 2022.
Outcomes	The primary outcome was all-cause mortality, and the secondary outcomes were 4-point major adverse cardiac events (MACEs) and major adverse kidney events (MAKEs), 4-point MACEs were defined as stroke (cerebral infarction or hemorrhagic stroke), acute myocardial infarction, cardiac arrest, and mortality, and 4-point MAKEs were defined as redialysis, dialysis dependence, estimated glomerular filtration rate (eGFR) < 15 ml/min/1.73 m2 and mortality.

Plants

Seed stocks	*Report on the source of all seed stocks or other plant material used. If applicable, state the seed stock centre and catalogue number. If plant specimens were collected from the field, describe the collection location, date and sampling procedures.*
Novel plant genotypes	*Describe the methods by which all novel plant genotypes were produced. This includes those generated by transgenic approaches, gene editing, chemical/radiation-based mutagenesis and hybridization. For transgenic lines, describe the transformation method, the number of independent lines analyzed and the generation upon which experiments were performed. For gene-edited lines, describe the editor used, the endogenous sequence targeted for editing, the targeting guide RNA sequence (if applicable) and how the editor was applied.*
Authentication	*Describe any authentication procedures for each seed stock used or novel genotype generated. Describe any experiments used to assess the effect of a mutation and, where applicable, how potential secondary effects (e.g. second site T-DNA insertions, mosiacism, off-target gene editing) were examined.*
